# Supplementary material for: The F-pilus biomechanical adaptability accelerates conjugative dissemination of antimicrobial resistance and biofilm formation
Source: Nat Commun. 2023 Apr 5;14:1879. doi: 10.1038/s41467-023-37600-y (PMC10076315; doi:10.1038/s41467-023-37600-y)
Supplement: Supplementary file 1 — Supplementary Information [file 41467_2023_37600_MOESM1_ESM.pdf]

SUPPLEMENTARY INFORMATION

The F-pilus biomechanical adaptability accelerates conjugative dissemination of antimicrobial resistance and biofilm formation

Jonasz B. Patkowski, Tobias Dahlberg, Himani Amin, Dharmender K. Gahlot, Sukhithasri Vijayrajratnam, Joseph P. Vogel, Matthew S. Francis, Joseph L. Baker, Magnus Andersson, and Tiago R. D. Costa

Supplementary Figures 1 to 5

Supplementary Tables 1 and 2

SUPPLEMENTARY FIGURES

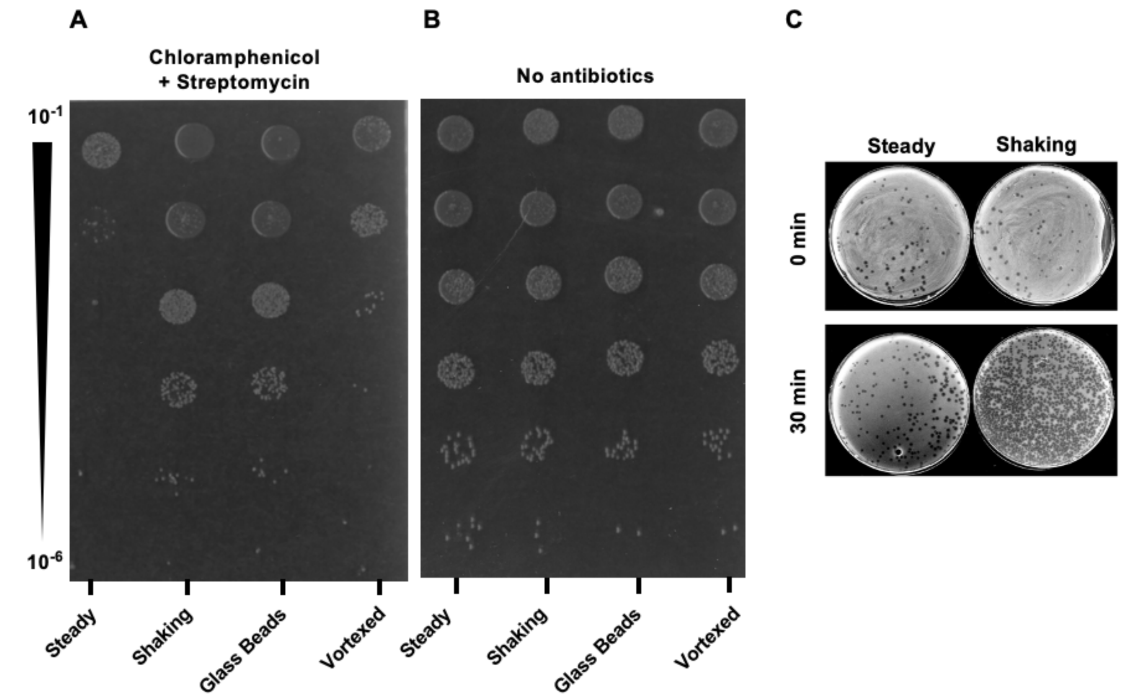

Supplementary Figure 1. Conjugation and viability assays. (A) Picture of a typical plate generated during the conjugation assay. (B) Picture of a typical plate generated during the accessory viability assay, showing that tested conditions do not affect donor and recipient cells viability. Gradient from 10^{-1} to 10^{-6} denotes serial. (C) Plaque formation by the f1 phage in steady and shaking conditions, showing higher phage attachment rate upon better mixing. Each experiment was repeated separately a minimum of three times.

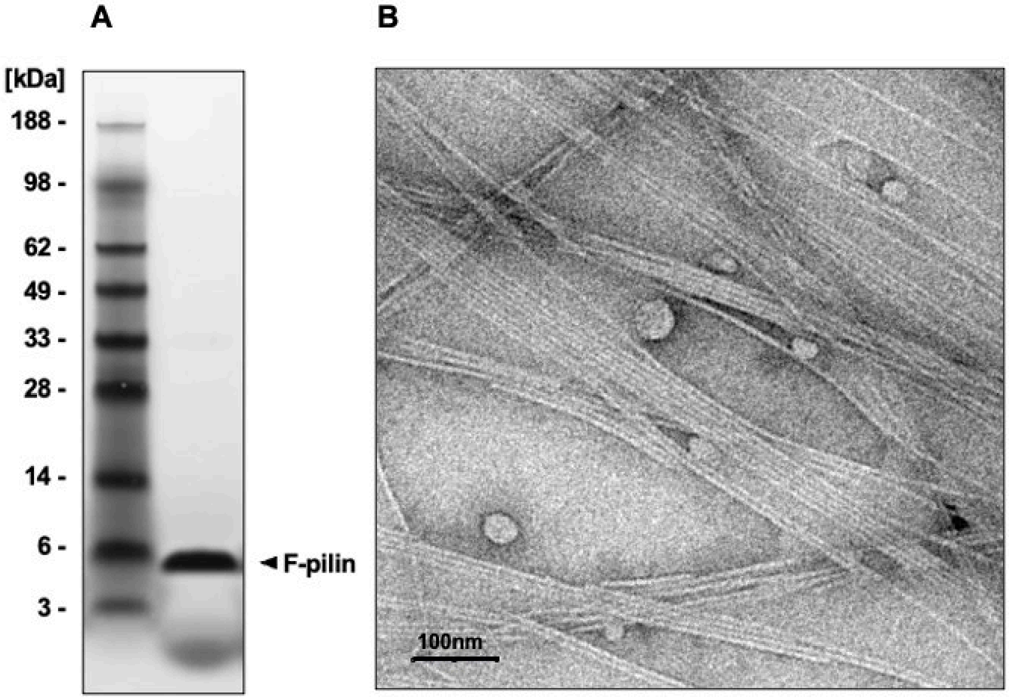

Supplementary Figure 2. Purification of F-pili (A) Picture of a typical SDS-PAGE gel of a purified fraction. (B) Negative stain Electron Microscopy image of the purified F-pili. The presence of F-pilin was further confirmed by LC-MS/MS. Each experiment was repeated separately a minimum of three times.

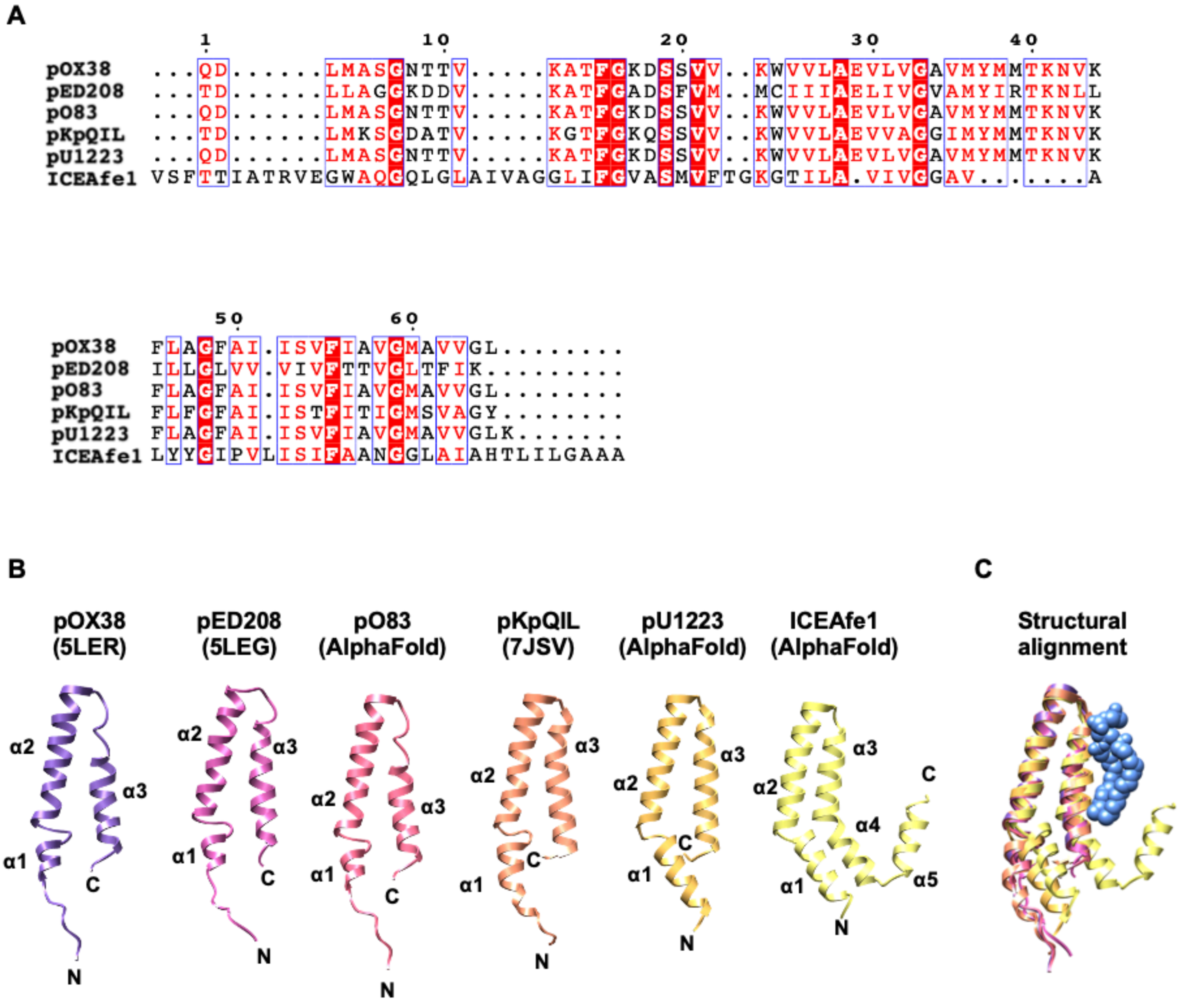

Supplementary Figure 3. F-pilin diversity encoded by the IncF plasmids. (A) Sequence alignment of the gene encoding the F-pilin from six different representative F systems. That includes the canonical *E.coli* K-12 pOX38 system (MF370216.1), *Salmonella typhimurium* pED208 (AF411480.1), *E.coli* O83:H1 pO83 (NC_017659.1), *Klebsiella pneumoniae* pKpQIL (NC_014016.1), *E.coli* urinary isolate pU1223 (SRR15011177), *Acidithiobacillus ferrooxidans* genome (NC_011761.1). The alignment was generated by ClustalW and visualized with ESPript. (B) Comparison of F-pilin structures, either solved experimentally (PDB ID given) or generated using AlphaFold. (C) Superimposition of the structures of the F-pilins with an interacting phospholipid molecule (shown in blue).

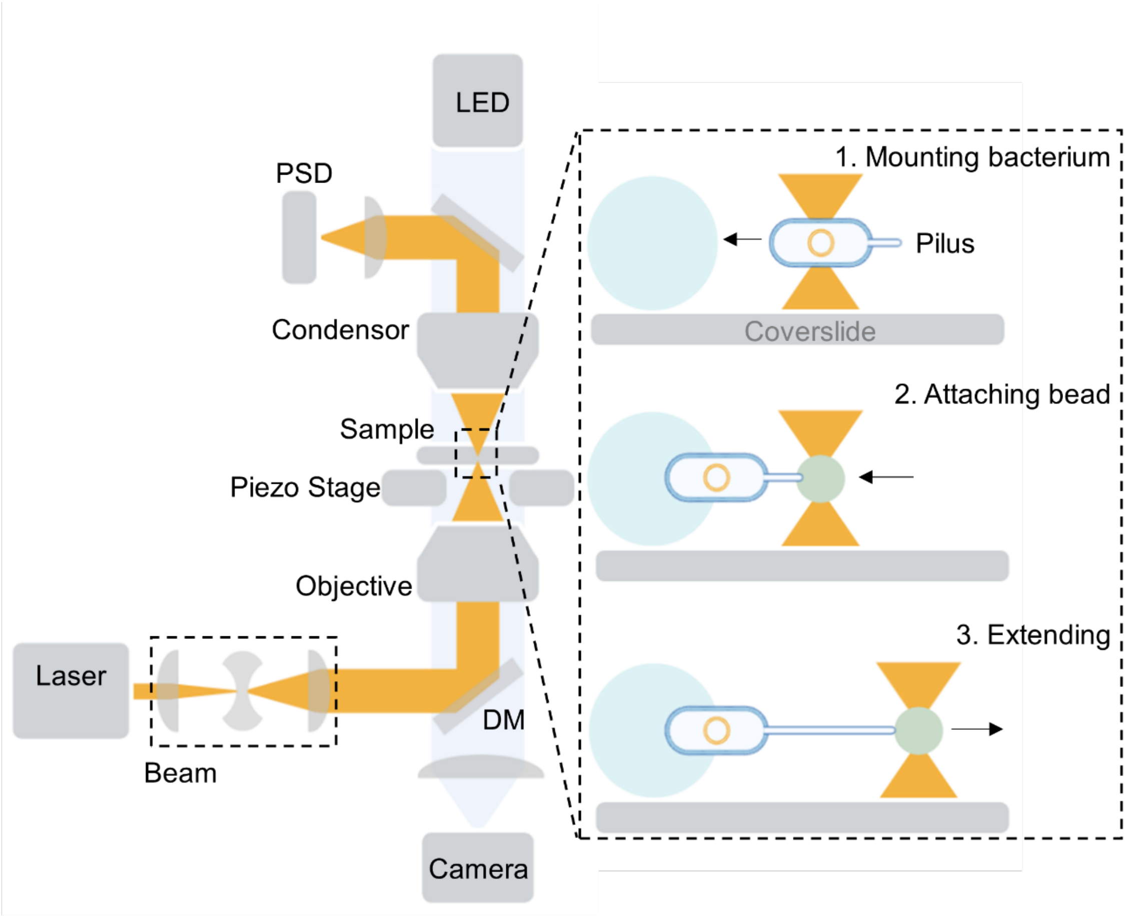

Supplementary Figure 4. Schematic of the optical tweezers setup. The inset shows the force measurement procedure. In (1) we trap and mount a piliated bacterium to a large poly-L-lysine coated microbead fixated to the sample coverslide. In (2) we trap and attach a small polystyrene microbead to a pilus expressed on the bacterium and in (3) we separate the trap and fixed bacterium to measure the pilus force-extension response. Figure cartoons were created with BioRender.com.

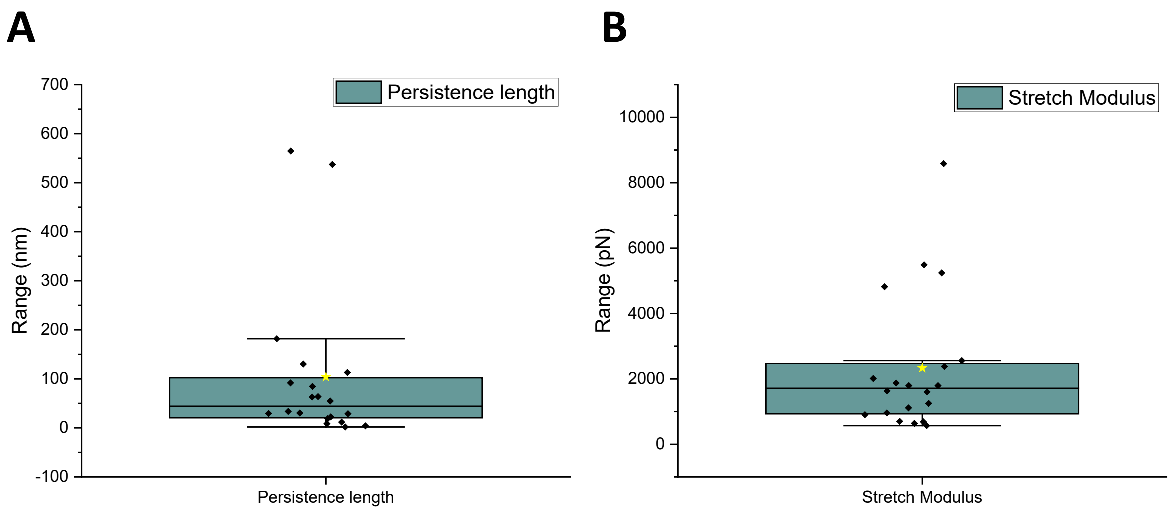

Supplementary Figure 5. The box plots show the 1st and 3rd quartiles (box limits), mean values (yellow stars), median values (solid lines), and 1.5·IQR (whiskers) for the A) persistence length and B) the stretch modulus. n=20 biological independent samples. Source data are provided as a Source Data file.

SUPPLEMENTARY TABLES

Supplementary table 1. Percentage of secondary structure content of simulated F-pilus models

Secondary structure type	Initial structure	Equilibration with lipids	Equilibration without lipids	Steered MD with lipids	Steered MD without lipids
**Extended**	0	0	0	0	0
**Bridge**	0	0	0	0	0
**3-10 helix**	0	6.2	5.8	6.7	4.4
**Alpha helix**	57.1	64.0	60.6	63.8	62.3
**Pi helix**	0	0	0	0	0
**Turn**	12.7	9.6	11.5	9.4	11.7
**Bend**	13.9	9.0	9.2	8.6	8.7
**Random**	16.3	11.2	12.9	11.5	12.9
**Total helical (sum of 3-10, Alpha, Pi)**	57.1	70.2	66.4	70.5	66.7

Supplementary table 2. Percentage of time for salt-bridge presence

	Glu29-Lys41	Asp18-Lys64
**Equilibration with lipids**	90.0	14.6
**Equilibration without lipids**	72.9	12.0
