# Supplementary material for: The F-pilus biomechanical adaptability accelerates conjugative dissemination of antimicrobial resistance and biofilm formation
Source: Nat Commun. 2023 Apr 5;14:1879. doi: 10.1038/s41467-023-37600-y (PMC10076315; doi:10.1038/s41467-023-37600-y)
Supplement: Supplementary file 3 — Description of Additional Supplementary Files [file 41467_2023_37600_MOESM3_ESM.pdf]

Description of Additional Supplementary Files:

Supplementary Movie 1. Simulation trajectory for each of the five sMD simulations for the F-pilus with phospholipids. The protein is shown in a cartoon representation, and the phospholipids are shown in a Van der Waals sphere representation.

Supplementary Movie 2. Simulation trajectory for each of the five sMD simulations of the F-pilus without phospholipids. The protein is shown in a cartoon representation.

Supplementary Movie 3. Simulation trajectory for the 100 ns equilibrium simulations of the F-pilus with and without phospholipids. The protein is shown in a cartoon representation, and the phospholipids are shown in a Van der Waals sphere representation.
