# Supplementary material for: The F-pilus biomechanical adaptability accelerates conjugative dissemination of antimicrobial resistance and biofilm formation
Source: Nat Commun. 2023 Apr 5;14:1879. doi: 10.1038/s41467-023-37600-y (PMC10076315; doi:10.1038/s41467-023-37600-y)
Supplement: Supplementary file 7 — Reporting Summary [file 41467_2023_37600_MOESM7_ESM.pdf]

nature portfolio

Corresponding author(s): Tiago Costa

Last updated by author(s): Mar 18, 2023

Reporting Summary

Nature Portfolio wishes to improve the reproducibility of the work that we publish. This form provides structure for consistency and transparency in reporting. For further information on Nature Portfolio policies, see our Editorial Policies and the Editorial Policy Checklist.

Statistics

For all statistical analyses, confirm that the following items are present in the figure legend, table legend, main text, or Methods section.

n/a	Confirmed	
☐	☒	The exact sample size (n) for each experimental group/condition, given as a discrete number and unit of measurement
☐	☒	A statement on whether measurements were taken from distinct samples or whether the same sample was measured repeatedly
☐	☒	The statistical test(s) used AND whether they are one- or two-sided<br>*Only common tests should be described solely by name; describe more complex techniques in the Methods section.*
☐	☒	A description of all covariates tested
☐	☒	A description of any assumptions or corrections, such as tests of normality and adjustment for multiple comparisons
☐	☒	A full description of the statistical parameters including central tendency (e.g. means) or other basic estimates (e.g. regression coefficient) AND variation (e.g. standard deviation) or associated estimates of uncertainty (e.g. confidence intervals)
☐	☒	For null hypothesis testing, the test statistic (e.g. F, t, r) with confidence intervals, effect sizes, degrees of freedom and P value noted<br>*Give P values as exact values whenever suitable.*
☒	☐	For Bayesian analysis, information on the choice of priors and Markov chain Monte Carlo settings
☒	☐	For hierarchical and complex designs, identification of the appropriate level for tests and full reporting of outcomes
☒	☐	Estimates of effect sizes (e.g. Cohen's d, Pearson's r), indicating how they were calculated

Our web collection on statistics for biologists contains articles on many of the points above.

Software and code

Policy information about availability of computer code

Data collection	Amber20/AmberTools21, MotionCor2
Data analysis	GraphPad Prism 7, MATLAB 2020b, AlphaFold2

For manuscripts utilizing custom algorithms or software that are central to the research but not yet described in published literature, software must be made available to editors and reviewers. We strongly encourage code deposition in a community repository (e.g. GitHub). See the Nature Portfolio guidelines for submitting code & software for further information.

Data

Policy information about availability of data

All manuscripts must include a data availability statement. This statement should provide the following information, where applicable:

- Accession codes, unique identifiers, or web links for publicly available datasets
- A description of any restrictions on data availability
- For clinical datasets or third party data, please ensure that the statement adheres to our policy

Models of F-pili were obtained from submitted PDB structures and PDB accession codes were specified where applicable. Sequences of F-pilins used were specified with GeneBank accession codes. All other experimental data used was generated for the purpose of this manuscript and not reported elsewhere. Raw datasets are available as a source data file.

Human research participants

Policy information about studies involving human research participants and Sex and Gender in Research.

Reporting on sex and gender	N/A
Population characteristics	N/A
Recruitment	N/A
Ethics oversight	N/A

Note that full information on the approval of the study protocol must also be provided in the manuscript.

Field-specific reporting

Please select the one below that is the best fit for your research. If you are not sure, read the appropriate sections before making your selection.

☒ Life sciences ☐ Behavioural & social sciences ☐ Ecological, evolutionary & environmental sciences

For a reference copy of the document with all sections, see nature.com/documents/nr-reporting-summary-flat.pdf

Life sciences study design

All studies must disclose on these points even when the disclosure is negative.

Sample size	For conjugation experiments, the sample size was n=6, and was regarded as 6 separate replicates using 6 separate bacterial colonies, as stated in Materials and Methods.<br>For biofilm formation measurements, the sample size was n=3 and included 3 biological replicates (3 separate bacterial colonies) followed by 3 absorbance measurements for each replicate, totaling to 9 measurements, as stated in Materials and Methods.<br>We determined the sample sizes based on preliminary studies in our laboratory or in similarly published research. The samples were enough to be detected and we observed statistical significant difference in replicated independent experiments.
Data exclusions	No data was excluded.
Replication	Each experiment was repeated at least three times independently with similar results.
Randomization	Bacterial colonies were picked by random. All samples were analyzed equally with no subsampling, and therefore was no requirement for randomization.
Blinding	Morphology of bacterial colonies was equivalent and they were picked by random.

Reporting for specific materials, systems and methods

We require information from authors about some types of materials, experimental systems and methods used in many studies. Here, indicate whether each material, system or method listed is relevant to your study. If you are not sure if a list item applies to your research, read the appropriate section before selecting a response.

Materials & experimental systems

n/a	Involved in the study	
☒	☐	Antibodies
☒	☐	Eukaryotic cell lines
☒	☐	Palaeontology and archaeology
☒	☐	Animals and other organisms
☒	☐	Clinical data
☒	☐	Dual use research of concern

Methods

n/a	Involved in the study	
☒	☐	ChIP-seq
☒	☐	Flow cytometry
☒	☐	MRI-based neuroimaging
